# African Swine Fever Virus Protein–Protein Interaction Prediction

**DOI:** 10.3390/v16071170

**Published:** 2024-07-20

**Authors:** Jacob A. Fenster, Paul A. Azzinaro, Mark Dinhobl, Manuel V. Borca, Edward Spinard, Douglas P. Gladue

**Affiliations:** 1 Oak Ridge Institute for Science and Education (ORISE), Oak Ridge, TN 37830, USA; jacob.fenster@usda.gov; 2 Plum Island Animal Disease Center, Foreign Animal Disease Research Unit, Agricultural Research Service, U.S. Department of Agriculture, Orient, NY 11957, USA; paul.azzinaro@usda.gov (P.A.A.); mark.dinhobl@usda.gov (M.D.); edward.spinard@usda.gov (E.S.); 3 National Bio and Agro-Defense Facility, Foreign Animal Disease Research Unit, Agricultural Research Service, U.S. Department of Agriculture, Manhattan, KS 66502, USA

**Keywords:** African swine fever virus, ASFV, African swine fever, ASF, protein–protein interactions, AlphaFold, genome-wide computational screen

## Abstract

The African swine fever virus (ASFV) is an often deadly disease in swine and poses a threat to swine livestock and swine producers. With its complex genome containing more than 150 coding regions, developing effective vaccines for this virus remains a challenge due to a lack of basic knowledge about viral protein function and protein–protein interactions between viral proteins and between viral and host proteins. In this work, we identified ASFV-ASFV protein–protein interactions (PPIs) using artificial intelligence-powered protein structure prediction tools. We benchmarked our PPI identification workflow on the Vaccinia virus, a widely studied nucleocytoplasmic large DNA virus, and found that it could identify gold-standard PPIs that have been validated in vitro in a genome-wide computational screening. We applied this workflow to more than 18,000 pairwise combinations of ASFV proteins and were able to identify seventeen novel PPIs, many of which have corroborating experimental or bioinformatic evidence for their protein–protein interactions, further validating their relevance. Two protein–protein interactions, I267L and I8L, I267L__I8L, and B175L and DP79L, B175L__DP79L, are novel PPIs involving viral proteins known to modulate host immune response.

## 1. Introduction

African swine fever (ASF) caused by the African swine fever virus (ASFV) is a devastating hemorrhagic disease of wild boars and domestic pigs that can be marked by high mortality rates. Since it was first described in 1920, there have been very few isolated outbreaks outside of Africa; however, in 2007, an outbreak occurred in Georgia that has since spread through Europe, Asia, and to the island of Hispaniola in the Western Hemisphere, causing a pandemic for ASF outbreaks. In 2023, Vietnam approved the commercial usage of a live attenuated vaccine based on the progenitor strain of the 2007 outbreak, ASFV Georgia 2007/1. Still, it is not fully understood if this vaccine would provide immunity against other ASFV variants that continue to cause outbreaks in Africa. To date, no subunit or vectored vaccine has been developed, and so, understanding the biology of ASFV virulence and immune correlates of protection are necessary for any subunit or vectored vaccine, and information, including how viral proteins interact with themselves and the host to replicate and spread, is central to creating affordable next-generation vaccines for this disease. These protein–protein interactions (PPIs) can provide insight at a molecular level as to how the virus is causing disease and replicating in the host. Further, they can provide insight into which gene or gene pairs may need to be deleted in combination to create attenuated strains harboring multiple deletions.

Many high-throughput methods exist for identifying PPIs in a chosen host organism. Experimentally, a yeast two-hybrid, (Y2H) and affinity-purification mass spectrometry (AP-MS), amongst other high-throughput techniques, have identified PPIs across all kingdoms of life, yet these methods suffer from extensive hands-on time, high cost, and significant false positive and false negative rates. In addition, the majority of identified sets of protein–protein interactions vary between Y2H and AP-MS methodologies. PPIs identified with these high-throughput techniques still require verification on the bench with in vitro studies. Proteome-wide computational techniques for identifying interacting proteins are desirable for organisms lacking experimental results as they could provide biological insight in a rapid and inexpensive fashion. The field of computational protein–protein interaction prediction is vast, as shown in the reviews in [1,2]. Successful proteome-wide computational PPI screening approaches to date have relied on one or a combination of (amongst other less critical data sources) (1) homologous complexes of known structure as templates to infer binding interfaces [3], (2) deep multiple sequence alignments, MSAs, of protein orthologs to extract co-evolutionary signals between protein chains, which identify binding interfaces [4,5], and (3) deep-learning-based protein structure prediction tools that require far lower quality MSAs and structural homology-based inputs for accurate prediction [6,7,8,9].

Applying computational proteome-wide PPI screening methods to viruses is especially tricky due to the rapid evolutionary rates and vastly different proteome compositions across viral taxa compared to cellular organisms. This diversity results in many viral proteins having no known homologs and shallow MSAs [10]. Experimental screens for YTH can be performed under BSL-2 conditions, but AP-MS requires contained BSL3 labs. As deep-learning-based protein structure prediction tools have been shown to infer accurate structures for protein monomers and complexes, even with low-quality MSA inputs, these tools could provide a route to discovering PPIs in the viral kingdom [6,11,12,13,14]. Indeed, AlphaFold2 PPI models have been used as an additional line of evidence for identifying a potential ASFV entry–fusion complex [15]. Here, we study ASFV PPIs to guide further research in virus–virus protein–protein interactions.

In this work, we have built on the success of the Humphreys et al. 2021 [6] pipeline to develop a workflow to screen for novel PPIs in nucleocytoplasmic large DNA viruses, NCLDVs. To address the issue of low sequence diversity of viral proteins, we have curated a custom protein database containing all complete viral genomes to add viral strain diversity in addition to species diversity currently present in homology searches. We then show that using the Humphreys et al. 2021 [6] lightweight AlphaFold complex prediction script outperforms the RoseTTAFold-2track model when using the low-diversity pMSAs as model inputs. This lightweight AlphaFold script was fast enough to screen all pairwise PPIs in the Vaccinia virus as a benchmark, as well as our experimental target, ASFV. High-scoring PPIs from this lightweight AlphaFold script were then analyzed with the full AlphaFold Multimer v2.3.2 model to provide further confidence in the potential biological relevance of the identified PPIs. Finally, we provide a discussion on the novel ASFV PPIs that we have identified with our workflow, with the goal of providing insights into future research using these predicted PPIs.

## 2. Materials and Methods

Our procedure for conducting a de novo screen to identify virus–virus PPIs was adapted from Humphreys et al. 2021 [6] and is described below.

### 2.1. Construction of a Strain-Diverse Viral Proteome Database 

As viruses evolve at an increased rate versus cellular organisms, a custom viral protein database was constructed to capture more viral strain diversity when searching for protein homologs. Our database includes the proteome from each complete viral genome deposited in the NCBI and, therefore, contains all deposited isolates of each given viral species. Only complete viral genomes were added to aid in protein pairing between peptides present in the same organism.

First, all complete viral genome accession numbers were retrieved with NCBI’s ESearch API tool by querying the nucleotide database with the following search term: “Viruses[Organism] + NOT + cellular + organisms[ORGN] + NOT + wgs[PROP] + NOT + gbdiv + syn[PROP] + AND + (srcdb_refseq[PROP] + OR + nuccore + genome + samespecies[FILTER] + OR + complete + genome[TITL])”. This returned 3,097,264 accession numbers (accessed November 2023). Next, NCBI’s EFetch API was used to retrieve the amino acid sequences annotated within each complete viral genome in the nucleotide database by specifying the retrieval mode “rettmode = fasta_cds_aa”. This database was cleaned to remove any amino acid sequences that contained “X”, “−“, “J”, “B”, or “Z” residues. The resulting database contains proteins from 1,682,927 viral genomes and 20,004,991 total viral proteins.

### 2.2. Viral Homolog Search

This custom database in Section 2.1 was searched for each viral protein query with JackHMMER v3.4 [16] using three iterations and otherwise default settings. The accession numbers of the Georgia 2007/1 (NCBI FR682468.2) and Vaccinia Virus WR are UniProt proteome ID UP000000344 from genome assembly GCA_000860085.1.

Next, the full database default MSA generation pipeline from AlphaFold v2.3.2 Multimer [14] was used to find additional homologs across all kingdoms of life. This includes a JackHMMER search of MGnify [17], a JackHMMER search of UniRef90 [18], an HHBlits [19] search of Uniclust30 [20], and BFD. As described in Section 1.2.2 in the Supplementary Information by Jumper et al. 2021 [11], the number of hits was limited to 5000 sequences for the JackHMMER search of MGnify, and there was a 10,000 hit limit for the JackHMMER search of UniRef90. HHBlits searches did not have limits. The default settings were used, except for the following flags during search for the following tools: JackHMMER: -N 1 -E 0.0001--incE 0.0001 --F1 0.0005 --F2 0.00005 --F3 0.0000005; HHBlits: -n 3 -e 0.001 -realign_max 100000 -maxfilt 100000 -min_prefilter_hits 1000 -maxseq 1000000.

The resulting local alignments for each database search were individually cleaned using the four steps below adapted from Humphreys et al. 2021 [6] and are implemented in the order presented.

1. Local alignment search tools, like JackHMMER, can return multiple local hits of the same template protein for a given query protein. One example of why this can occur is protein domain swap events. Multiple local hits of the same protein are merged by concatenation in the same order as the viral protein alignment. If any overlapping regions between the template alignments exist, the overlapping alignment section with a higher percent ID to the query alignment section is taken.
2. Multiple distinct protein hits from a single viral proteome can occur for a given protein query. When this occurs, the protein with the highest percent ID alignment (identical columns divided by aligned columns to query) is taken, and all other protein hits from the given proteome are discarded.
3. Columns that are gaps in the query sequence are deleted from all alignments.

Any alignment with a gap score (fraction of gapped columns in the target alignment divided by the query length) below 0.5 was discarded. Homolog sequences from the five alignments were de-aligned and combined for each viral protein query. See Supplementary Figure S8 for a flowchart representation of the viral homolog search. 

### 2.3. Multiple Sequence Alignment Generation 

Each set of viral protein homologs generated in Section 2.2 was aligned using the HMMER v3.4 seed alignment algorithm presented in Humphreys et al. 2021 [6].

Briefly, Phmmer is used to search a viral protein query against its homologs using the default search parameters. The search result MSA is filtered to keep alignments most like the query alignment to create a “seed” alignment. This “seed” alignment is converted to a Hidden Markov Model (HMM) with HMMbuild and used to align the original set of viral homologs using HMMalign. 

In more detail, the results of the default Phmmer search of the original query sequence versus its combined viral homologs are cleaned using step 1 in Section 2.2 to merge duplicate local alignments for the same protein homolog. The alignments from this cleaned Phmmer search are filtered using three independent sets of criteria: (1) a gap ratio of <0.2 and a sequence identity of >0.55, (2) a gap ratio of <0.35 and a sequence identity of >0.4, (3) and a gap ratio of <0.5 and an identity of >0.25. Here, the gap ratio is defined as the number of gaps in the target protein that fall in query protein columns divided by the length of the query protein. The sequence identity is defined here as the number of identical columns between the target and query divided by the number of aligned columns (columns where both query and target have amino acids). The most stringent filtering criterion that can select over 2500 or 25% of all sequences of the homologous group is used, and this group of alignments is selected as a “seed” alignment. This “seed” alignment is converted into an HMM with HMMbuild using the default parameters. This HMM is used to align the original list of viral homologs using HMMalign with the default parameters. The alignments generated with HMMalign are then cleaned using steps 2 and 3 in Section 2.2 to yield a combined viral homolog MSA. See Supplementary Figure S8 for a flowchart representation of the multiple sequence alignment generation.

### 2.4. Paired MSA (pMSA) Generation, Heterodimers

For each pair of different viral proteins, protein A and protein B, we generated paired multiple sequence alignments as follows from their corresponding combined homolog search MSAs from Section 2.4:

1. Alignments that originated from the custom viral complete genome database described in Section 2.1 were paired (concatenated) if they both existed in the same viral proteome. This represents the paired section of the pMSA; see Supplementary Figure S1.
2. All other homolog alignments (unpaired homolog alignments from the custom viral complete genome database and homolog alignments from the AlphaFold MSA generation pipeline) were not paired and were kept as unpaired alignments corresponding to protein A or protein B; see Supplementary Figure S1.
3. The paired alignments were filtered at 99% sequence ID with HHfilter v3.3.0 [21]. The two unpaired alignments corresponding to protein A and protein B were filtered at 90% sequence identity with HHfilter v3.3.0.
4. The filtered unpaired alignments from protein A and protein B were then compared to the paired alignments. Any identical matches between the unpaired protein A alignments and the protein A region of the paired alignment were removed from the unpaired protein A alignments. Likewise, any identical matches between the unpaired protein B alignments and the protein B region of the paired alignments were removed from the unpaired protein B alignments.
5. pMSAs were then assembled from the three filtered alignment groups. Paired alignments were simply added to the pMSA. Unpaired protein A alignments had gaps added to the columns corresponding to protein B. Likewise, unpaired protein B alignments had gaps added to the columns corresponding to protein A.

These pMSAs were used as heterodimer pMSA inputs for the HAF model described in Section 2.8 below. When screening with RoseTTAFold, as shown in Section 2.7, only the paired alignments were extracted from the pMSAs and used as inputs, omitting the unpaired Section of the pMSA, as previously described [6]. 

We omitted the long ASFV proteins CP2475L, NP1450L, G1340L, M1249L, EP1242L, G1211R, P1192R, D1133L, F1055L, C962R, B962L, and A859L from all analysis to speed up the computation time. See Supplementary Figure S9 for a flowchart representation of the heterodimer pMSA generation.

### 2.5. Paired MSA Generation, pMSAs, and Homodimers

For each viral protein homodimer pMSA, the first line of each pMSA was set to the duplicated protein query sequence. No other alignments were paired for homodimers. Instead, all combined homolog alignments in Section 2.3 were filtered at 90% sequence identity with HHfilter. Gaps were concatenated to the left and right side of this filtered homolog alignment to generate the unpaired Sections of the pMSA; see Supplementary Figure S1. For homodimers, unpaired alignments are the same for protA and protB. These pMSAs were used as homodimer pMSA inputs for the HAF model described in Section 2.8 below. See Supplementaty Figure S9 for a flowchart representation of the homodimer pMSA generation.

### 2.6. Calculating pMSA Diversity: Nseq_80_ Calculation

The number of sequences at 80% ID (Nseq_80_) in the pMSA were calculated as follows using a similar approach as described in Zhang et al. 2020 [22]. The pMSAs in Section 2.4 and Section 2.5 were separated back into three multiple sequence alignments corresponding to the paired alignments, unpaired protein A alignments, and unpaired protein B alignments, as shown in Supplementary Figure S1. For the protein A unpaired alignments, the gaps that filled the protein B columns were removed. Likewise, for the protein B unpaired alignments, the gaps that filled the protein A columns were removed. HHfilter v3.3.0 was then used to filter all three alignments at 80% ID. Nseq80 for the paired, unpaired protein A, and unpaired protein B were calculated by counting the number of resulting alignments after filtering each respective separated MSA. 

### 2.7. RoseTTAFold 2-Track Model Screening 

The RoseTTAFold v1.1.0-2track model described in Humphreys et al. 2021 [6] was used to screen all combinations of PPIs. All model weights and genetic databases were current releases as of October 2023. Briefly, this model uses a smaller model with 10.7 M parameters versus the full 130 M parameters in the full three-track RoseTTAFold model. This model was trained on the same 208,659 single protein chains released in the PDB as of 17 February 2020 as the full RoseTTAFold model. These structures were cropped at 300 residues to fit into GPU memory and input as “discontinuous crops” with chain breaks in training examples to facilitate PPI prediction performance. For more details on model benchmarking and details on the loss function used, see Humphreys et al. 2021 [6]. When predicting structures of protein–protein pairs, 200 was added to the residue number of the second protein chain to let the network know that there is a chain break between each subunit. 

The maximum contact probability score was calculated as described in Humphreys et al. 2021 [6]. Briefly, RoseTTAFold calculates the probability, termed contact probability, that two C_β_ atoms of each pairwise set of residues reside within 12 Å of one another. These data are formatted as a distance matrix in the .npz file output. To study the inter-chain contact probabilities, the subset of the full distance matrix representing contacts between the two protein chains is extracted. The contacts between the last 10 C-terminal residues of the first protein and the first 10 N-terminal residues of the second protein were excluded. From this set of contact probabilities, the maximum value was calculated, termed max contact probability. Within each virus PPI set, the average product correction was used according to Humphreys et al. 2021 [6] to penalize proteins that bind many other proteins and serve as false positive hubs. Equation (1), reproduced from Humphreys et al. 2021 [6], is shown below.

(1) $$\begin{array}{l} score_{X,Y}=prob_{X,\;Y}+prob_{X,\;Y}-\left(\sum^{\;}prob_{X,i}\right)\times \left(\sum^{\;}prob_{Y,\;j}\right)/\left(\sum^{\;}\sum^{\;}prob_{i,j}\right),\;ifprob_{X,\;Y}\geq 0.9\\ score_{X,Y}=prob_{X,\;Y}-\left(\sum^{\;}prob_{X,i}\right)\times \left(\sum^{\;}prob_{Y,\;j}\right)/\left(\sum^{\;}\sum^{\;}prob_{i,j}\right),\;ifprob_{X,\;Y}<0.9 \end{array}$$

where $prob_{X,\;Y}$ is the max contact probability of protein pair X and Y, $\sum^{\;}prob_{X,i}$ is the sum of max contact probabilities of protein X versus all other possible protein pairs, $\sum^{\;}prob_{Y,i}$ is the sum of max contact probabilities of protein Y versus all other possible protein pairs, and $\sum^{\;}\sum^{\;}prob_{i,j}$ is the sum of the max contact probabilities of all pairs in the viral PPI set. 

### 2.8. Humphreys AlphaFold Lite (HAF) Protein Complex Screening 

A singularity container was built to run AlphaFold v2.0.1 (https://github.com/prehensilecode/alphafold_singularity (accessed on 18 December 2023)) [11]. AlphaFold model parameters were updated as of 6 December 2022 and downloaded via https://storage.googleapis.com/alphafold/alphafold_params_2022-12-06.tar (accessed on 4 December 2022). All genetic and protein structure databases were the current releases of the databases downloaded in November 2023. The AlphaFold prediction script published in Humphreys et al. 2021 [6] was used to screen large numbers of protein–protein interactions, as this script reduces compute time versus a default full database run AlphaFold multimer by a factor of 10 [6]. Briefly, the Humphreys et al. 2021 [6] AlphaFold PPI prediction script runs AlphaFold v2.0.1, adds 200 to the residue number of the second protein chain to let the AlphaFold network know there is no chain connection between each protein pair, uses only AlphaFold models 1, 3, and 5 to predict the PPI structure, and accepts pre-computed pMSAs. In addition, this script skips the Amber relaxation step present in the full AlphaFold model. 

The maximum contact probability score was calculated as described in Humphreys et al. 2021 [6]. Briefly, AlphaFold calculates the probability, termed contact probability, that two C_β_ atoms of each pairwise set of residues reside within 12 Å of one another. These data are formatted as a distance matrix in the .npz file output. To study the inter-chain contact probabilities, the subset of the full distance matrix representing contacts between the two protein chains is extracted. The contacts between the last 10 C-terminal residues of the first protein and the first 10 N-terminal residues of the second protein were excluded. From this set of contact probabilities, the maximum value was calculated, termed max contact probability, and used for benchmarking and as a threshold for further validation in the full AlphaFold Multimer model. 

### 2.9. AlphaFold Multimer (AFmult) Validation 

A singularity container was built to run AlphaFold Multimer v2.3.2 (https://github.com/prehensilecode/alphafold_singularity (accessed on 20 October 2023)) [14]. This was used to validate high-scoring PPIs from the Humphreys AlphaFold lite script. The default MSA generation and template search pipelines were used to search the full genetic and structure databases of the current releases as of November 2023. The max template date was set to 1 January 2022, and two multimer predictions were calculated per model. The score used to asses AlphaFold Multimer model accuracy is the weighted combination of the predicted template modeling score (pTM) and the interface pTM score (ipTM) according to the equation below, which was taken from [14].

(2) model confidence=0.8∗ipTM+0.2∗pTM

### 2.10. Vaccinia Gold-Standard Set 

The Vaccinia virus was used as a viral model to benchmark the performance of the PPI identification pipeline presented in this work. The Molecular INTeraction Database, MINT, [23] was queried for experimentally determined Vaccinia virus–Vaccinia virus PPIs using a custom search script that looked for both proteins to either have the taxID 10254 or the name “Vaccinia” or “vaccinia”, “Vaccinia virus”, or “vaccinia virus”. PPIs from this search that were not of proteins present in the Vaccinia virus WR strain were mapped back to this isolate by a BLASTp search. Redundant PPIs were filtered. This resulted in a gold-standard set of 52 experimentally validated PPIs.

### 2.11. Performance Evaluation of the Vaccinia Virus PPI Screen

**Figure 1:**
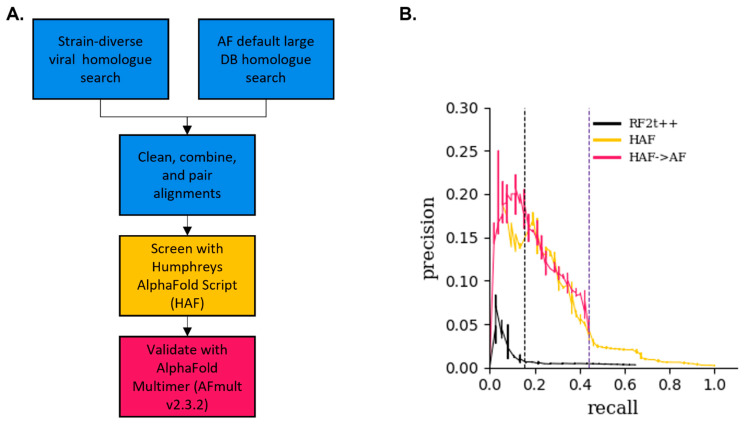
A novel Poxvirus PPI identification workflow using AlphaFold. (**A**) Flowchart of the pMSA generation and AlphaFold screening, as shown in the Materials and Methods Section 2.1, Section 2.2, Section 2.3, Section 2.4, Section 2.5, Section 2.6, Section 2.7, Section 2.8 and Section 2.9. A viral homolog search is conducted from a custom strain-diverse viral genome database and combined with the default AlphaFold homolog search. Alignments are cleaned and combined across search databases. Pairing only occurs between proteins present in the same viral genome. pMSAs are screened by the Humphreys et al. 2021 [6] AlphaFold v2.0 lightweight script, the HAF, to calculate the maximum contact probability; see Section 2.8. PPIs with higher than a 0.8 max contact probability are further validated on AphaFold Multimer v2.3.2, AFmult, using the default settings. For a more detailed workflow, see Supplementary Figures S8 and S9. (**B**) Precision–recall performance curve of various models on the Vaccinia virus. A total of 52 experimentally verified gold-standard PPIs extracted from the MINT database were assumed to be sole positive controls, and the 23,819 other possible PPIs were assumed to be negative controls; see Section 2.10 and 2.11. RF2t++, black line, is the RoseTTAFold two-track model with the average product correction. HAF, yellow line, is the Humphreys et al. 2021 [6] lightweight AlphaFold script. The HAF->AF, pink line, represents PPIs that scored ≥ 0.8 max contact probability on the HAF and were run through AlphaFold Multimer, while all other PPIs < 0.8 had max contact probability on the HAF set to zero. The right vertical purple dashed line at recall = 0.44 represents the threshold of 0.8 max contact probability applied to the HAF screen. Here, the recall is 0.44 and the precision is 0.04. The left vertical black dashed line at recall = 0.15 represents the threshold of 0.8 ipTM + pTM applied to the HAF->AF screen. Here, the recall is 0.15 and the precision is 0.19.

A precision–recall curve was generated from the genome-wide Vaccinia virus PPI computational screen, presented in Figure 1B. Positive control PPIs were taken from the Vaccinia gold-standard set, Section 2.10, and all other pairwise combinations of proteins were taken as negative controls. This resulted in 52 positive controls and 23,819 negative controls. The RF2t++ score, max contact probability, or ipTM + pTM score were, respectively, swept from 0 to 1, and the precision and recall were calculated at each threshold, as shown in Supplementary Figure S8. Precision is defined as the number of true positives called at a given threshold divided by the total number of calls at the same threshold. Recall is defined as the number of true positives called at a given threshold divided by the total number of true positives in the experiment. 

### 2.12. ASFV Gold-Standard Set

A recent review [24] cataloed all the experimental literature for ASFV-ASFV and ASFV–host protein–protein interactions. Nine ASFV-ASFV interactions were extracted from this dataset; see Supplementary Data. The MINT database search for ASFV-ASFV interactions using the search term [“asfv”, “ASFV”, “african swine fever virus”, “African swine fever virus”, “asfv”, “ASFV”, “african swine fever virus”, “African swine fever virus”, “African swine fever virus, ASFV”] OR taxID = 10,497 did not return any results. 

### 2.13. Data Analysis and Visualization 

Custom Python scripts were used for data analysis and most visualizations. Matplotlib was used for plotting line plots and histograms. BIOVIA Discovery Studio Visualizer 2024 was used for protein structure visualization and for identifying and labeling non-covalent interactions between predicted interacting proteins. The hydrophobicity surface plots were generated using BIOVIA’s solvent surface rendering using a 1.4 Angstrom probe radius. pDLLT confidence score visualization was conducted with NCBI’s iCn3D web-based structure visualizer, https://www.ncbi.nlm.nih.gov/Structure/icn3d/full.html (accessed on 25 April 2024). Pie charts were plotted in Microsoft Excel version 2405. 

## 3. Results

### 3.1. A Computational Workflow for PPI Identification in Nucleocytoplasmic Large DNA Virus 

#### 3.1.1. Strain-Diverse Viral pMSA Generation

Building off of the success of the Humphreys et al. 2021 [6] PPI identification pipeline in yeast, we developed a bioinformatic workflow that can be used to identify predicted PPIs in nucleocytoplasmic large DNA viruses, NCLDVs. The Humphreys et al. 2021 [6] workflow relied on the generation of pMSAs with high sequence diversity for the RoseTTAFold model to feature amino acid co-evolution signals between chains to guide predicting protein contact. We, therefore, focused on incorporating as much diversity as possible with the publicly available genetic databases. We started with OrthoDB, a large protein ortholog database containing 7962 viral genomes in its ortholog groupings [25]. After pulling all viral orthologous groups containing ASFV proteins, only 51 of the 193 ASFV proteins were represented in the orthologous groups. Of these 51 orthologous protein groups, only 21 groups had more than 10 unique sequences. This left only a handful of proteins that were above the cutoff of 200 paired sequences used in the Humphreys et al. 2021 [6] yeast work. We, therefore, attempted to increase our pMSA diversity by increasing our proteome database size and by lowering the threshold criteria for adding a sequence to a given alignment.

To expand the sequence diversity of virus–virus pMSAs, a custom viral protein database was constructed that pulled coding sequences (CDSs) from all complete viral genomes present on the NCBI nucleotide database, described in detail in Materials and Methods Section 2.1. This contrasts with many existing protein databases, which have only one or a few select representatives for each species. 

While grouping protein sequences into orthologous groups is preferred to paralog/homolog searches, as orthologs are more likely to have conserved functions, we decided to conduct homolog searches of the various databases for the following reasons: (1) clustering orthologous groups from large databases is a computationally expensive task, (2) viral orthologous groups are poorly defined due to the lack of similarity and host species specificity between viral species, and (3) homolog searches can be more sensitive than ortholog searches, as the reciprocal best hit criterion does not need to be satisfied. We ensured that only a single protein hit was selected per unique genome. This allowed us to pair viral homologs if both homologs were present in the same genome; see Materials and Methods Section 2.1, Section 2.2, Section 2.3, Section 2.4 and Section 2.5 for details on our pMSA generation workflow. 

#### 3.1.2. Benchmarking PPI Identification in the Vaccinia Virus

Both the Vaccinia virus and ASFV are NCLDVs that share a similar genome size and encode a similar number of genes. To benchmark various pipeline configurations, we used the Vaccinia virus because compared to the ASFV, there is a wealth of experimentally verified PPIs. For example, there are only eight reported ASFV-ASFV PPIs in the scientific literature [24] versus ninety-five Vaccinia–Vaccinia PPIs, including one comprehensive yeast two-hybrid screen performed with the Western Reserve (WR) strain, which was chosen as the reference proteome for PPI modeling in this work (proteome ID UP000000344). We constructed a gold-standard set of experimentally verified PPIs by extracting all Vaccinia–Vaccinia PPIs from the Molecular INTeraction Database (MINT) [23] and cross-referencing all sequences to the Vaccinia–WR proteome to remove redundant pairs. This resulted in 52 gold-standard Vaccinia–WR PPIs. Vaccinia virus pMSAs for all possible protein–protein interactions were constructed using an identical pipeline as the ASFV pMSAs.

Supplementary Figures S2 and S3 show pie charts of the viral phyla represented in the ASFV and Vaccinia virus homolog searches generated from our custom viral database, respectively. Supplementary Figures S4 and S5 show histograms of the number of paired sequences in our heterodimer pMSAs for the ASFV-ASFV heterodimers and the Vaccinia–Vaccinia heterodimers, respectively. In addition, Supplementary Figures S6 and S7 show histograms of our ASFV and Vaccinia virus pMSAs, respectively, clustered at 80% sequence identity, as this is a common metric for quantifying MSA sequence diversity [11]. Despite our efforts in maximizing the sequence diversity of the paired viral homologs, the average number of paired sequences in the ASFV and Vaccinia (12 and 56, respectively) is far below the 200-count cutoff implemented in the Humphreys et al. 2021 [6] RoseTTAFold screen. Regardless, we ran all 23,653 Vaccinia virus heterodimer pMSAs through the two-track RoseTTAFold model and RF2t++ using the APC correction described in Humphreys et al. 2021 [6]. A total of 23,603 of the runs were completed successfully, with 50 PPIs failing due to lack of sufficient RAM due to large protein size, and they were omitted from further analysis. To calculate model performance metrics, we take the 52-member gold-standard set as positive PPIs and we assume all other PPIs as negative PPIs. As our gold standard set is likely smaller than the true number of all binary PPIs in the Vaccinia virus, we interpret our benchmark to be a conservative estimate of true model performance. The RF2t++ model performed poorly, as shown by the black curve in Figure 1B. We, therefore, looked for another PPI prediction model that can produce a higher precision and recall with our low-diversity pMSA inputs. 

It has been shown that AlphaFold v2.0/v2.3.2 can often infer accurate monomeric structures with limited to no sequence diversity [11,26]. To see if AlphaFold can accurately predict PPIs with poor pMSA diversity input, we used the lightweight AlphaFold script presented in Humphreys et al. 2021 [6], hereafter called the HAF, described in Section 2.8. This script accepts a precompiled pMSA and reduces compute time using three out of the five AlphaFold monomer models by skipping the Amber relaxation step of the predicted PPI. With the knowledge that unpaired protein sequences can enhance protein structure prediction with AlphaFold, we added unpaired protein sequences obtained by homolog searches performed by the default AlphaFold MSA generation of the full genetic databases to our previously created pMSA. Figure 1A shows a flowchart of how the pMSAs were generated for the PPI identification pipeline. Supplementary Figures S8 and S9 show the bioinformatic workflow used in this paper in more detail.

All 23,653 Vaccinia virus heterodimer PPIs and 218 homodimer PPIs were modeled with the HAF. Each PPI model was scored by its maximum contact probability, the highest predicted probability that two C_β_ atoms from separate chains are within 12 Å of one another. The yellow curve in Figure 1B shows the performance of the HAF model on all hetero- and homodimers. This screen gives a precision of ~16% at a recall of 20%, which is significantly improved versus a precision of 0.7% at the same recall of 20% for the RF2t++ screen, as shown by the black curve in Figure 1B. 

We sought to further improve the precision of our screen by running all high-scoring PPIs from the HAF model (those with max contact probabilities ≥ 0.8) through the full AlphaFold Multimer v2.3.2 model, hereafter AFmult. While it would have been ideal to run all PPIs through AFmult, computational limitations due to significantly longer compute time versus the HAF prevented a comprehensive computational screen with AFmult. The AFmult model provides the interfacial predicted template modeling score (ipTM), an improved PPI model quality score versus max contact probability, and conducts an Amber relaxation step on the predicted protein structures for higher accuracy visualizations. Out of the 592 Vaccinia virus PPI models ran on AFmult, 572 models were successfully completed, while 20 models failed to run due to insufficient RAM due to large protein sizes and were discarded from future studies. The pink curve in Figure 1B shows the precision–recall of this two-stage screen and demonstrates that using AFmult to further validate high-scoring PPIs predicted from the HAF model results in an increase in precision at lower recall values, as shown in Figure 1B and Supplementary Figure S10. Based on this information, a threshold of 0.8 ipTM + pTM was chosen because it has good metrics with our Vaccinia virus benchmarking and because it has been previously reported to correlate accurate models (average DockQ score of ~0.7) [14]. At this threshold, our two-stage screen has a precision of 19% and a recall of 15%, as shown by the black dashed line in Figure 1B. Table 1 shows summary metrics of the Vaccinia virus computational PPI screen.

With these performance metrics in mind, we applied our identification pipeline to the ASFV Georgia 2007/1 (FR682468) strain. We modeled 18,913 heterodimers and 197 homodimers, of which 375 passed the initial HAF filter and 19 passed the AFmult threshold of 0.8 ipTM + pTM, as shown in Table 1. 

### 3.2. ASFV Identified PPIs 

Table 2 below shows the ASFV-ASFV protein–protein interactions identified with our two-stage, genome-wide, AlphaFold screen. Supplementary Figures S9 and S10 show the predicted structures of the nineteen PPIs that scored at or above 0.8 ipTM + ptm along with their AFmult pDLLT local confidence scores, B646L__B646L, with a HAF max contact probability of 0.76, which did not make the AFmult cutoff, but we ran this PPI through AFmult, as it is a gold-standard ASFV-ASFV interaction. While ASFV B66L__B66L and C717R__C717R scored above our AFmult significance cutoff, these two structures were omitted from further analysis because the validity of their predicted structures was not convincing. B66L likely contains a transmembrane domain, and the predicted dimer does not show an interaction between the region outside of the membrane; rather, the interacting region is the same alpha helix rotated 180 degrees, as shown in Supplementary Figure S12B. We interpret C717R__C717R as a symmetry artifact, and it was omitted from further analysis because the interacting non-covalent residues are sparse and there are many charged external facing residues that are not utilized in the protein–protein interface, as shown in Supplementary Figure S12D. We removed these two entries from Table 2.

#### 3.2.1. I267L__I8L 

I8L is a highly conserved gene in the ASFV [27,28]. The single knockout of I8L in ASFV Georiga 2007 does not affect virus replication in vitro or in vivo and does not change the disease outcome in swine [28]. Though, I7L-I11L (alternate name L7L-L11L) deletion in ASFV SY18 strain reduced virulence and offered protection against parental ASFV SY18 challenge [29]. I267L is experimentally determined to inhibit RNA Pol-III-RIG-I-mediated antiviral response by interacting with Riplet and preventing the activation of RIG-I [30]. Knockout of the I267L gene in ASFV SY18 did not affect its replication nor virulence, though similar deletion in ASFV CN/GS/2018 resulted in attenuated virulence and pathogenesis in pigs [30]. 

**Figure 2:**
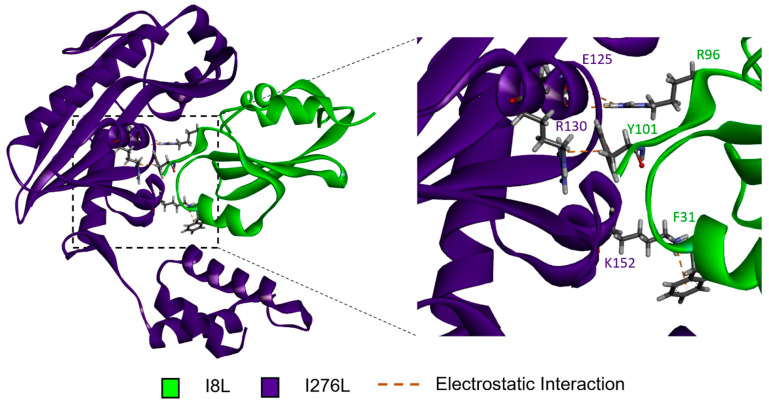
I267L__I8L predicted structure. I8L, green, and I276L, purple, are shown as ribbon structures. Residues predicted to engage in favorable electrostatic non-covalent interactions are shown as stick representations, with orange dashed lines representing the favorable electrostatic interaction. The boxed area on the left is zoomed in on the right to better see the highlighted interactions. The residues predicted to interact are labeled and color coded—green for I8L and purple for I276L.

Figure 2 shows the AFmult predicted structure of I267L__I8L. Favorable electrostatic non-covalent interactions dominate the favorable interactions on the protein–protein interface. 

#### 3.2.2. CP530R__S273R

pS273R, a cysteine protease, has been experimentally shown to process the polyprotein product of CP530R, p62, into the products p8 and the previously crystallized proteins p35 and p15, which are part of the core–shell structure [31,32,33,34]. The cleavage occurs at the consensus sequence Gly-Gly-X to produce the major structural proteins p35 and p15 [33]. Indeed, the predicted AFmult structure places the experimentally determined catalytic pS273R CYS 232 within 7 Å from CP530R’s experimentally determined Gly 158, Gly 159, Asn 160, and cleavage site, as shown in Supplementary Figure S13 [32]. The interaction between pS273R and p62 seems to be dominated by non-covalent electrostatic amino acid interactions, as shown in Supplementary Figure S13. 

#### 3.2.3. F334L__F778R

The cytoplasm of host cells serves as the site for DNA replication in NCLDVs. Often, a viral factory is formed with a large number of viral proteins; hence, the viral genome encodes many of the proteins essential for DNA synthesis, as the replication enzymes of the host are primarily confined to the nucleus; however, some host proteins are likely recruited to the viral factory. F334L and F778R are the predicted small and large subunits of the ribonucleotide reductase complex, which synthesized precursors needed for DNA synthesis, and they are highly similar to the small and large ribonucleotide reductase subunits in the Vaccinia and Orthopoxviurs [35] and are well represented across the viral kingdom. The F334L__F778R pMSA generated here has a paired Nseq80 (number of sequences clustered at 80% sequence identity) value of 450. 

#### 3.2.4. MGF PPIs

The ASFV encodes groups of proteins that exhibit common conserved motifs and can be categorized into five multigene families (MGFs) based on the average length of the amino acids encoded by the genes belonging to each family: MGF 100, MGF 110, MGF 300, MGF 360, and MGF 505 [36]. Genes within the MGF families are believed to modulate the host immune response. Still, while attenuated strains have been demonstrated to contain deletions of genes from multiple MGFs, other isolates, such as Estonia 2014, RV502, and Ghana2022-35, which contain a loss of MGF genes, have been sequenced from ASF outbreaks, indicating that the role of MGF genes during infection is complicated. Further, in vivo and in vitro studies have demonstrated that targeted deletion of certain genes within the MGF 360 family can result in the inability to replicate in tick, while replication was unaffected in primary porcine macrophage, indicating that MGF genes may be linked to host specificity [37]. 

#### 3.2.5. MGF_360-1La__MGF_360-1Lb

MGF_360-1L (also known as KP306L) has been recently shown to not be required for replication in primary porcine macrophages or virulence during infection in swine [38]. In most genotypes, MGF_360-1L is translated as a single protein; however, in most ASFV Georgia 2007/1 variants, a deletion of a single C within codon 100 results in a frameshift and early truncation of MGF_360-1L. Accordingly, MGF_3601L has been annotated as MGF_360-1Lb and MGF_360-1La and has been demonstrated to be transcribed on a single bicistronic [39] transcript. Our data indicate that despite being split into two proteins, MGF_360-1La/1Lb can still complex via favorable hydrophobic interactions to form a multimer, assuming both proteins are translated, as shown in Supplementary Figure S14A. In addition, the C termini of MGF_360_1Lb and the N termini of MGF_360-1La are predicted to reside within 16.5 Å of one another, as shown in Supplementary Figure S14A. 

#### 3.2.6. MGF_360-19Ra__MGF_360-19Rb

Surprisingly, although there is limited information available regarding MGF_360-19 in comparison to MGF_360-1L, similar to MGF_360-1L, it has been observed that historical ASFV isolates typically encode MGF_360-19R as a single protein. Moreover, strains that resemble ASFV Georgia 2007/1 have MGF_360-19R annotated as two ORFs. Our AFmult models predict that ASFV Georiga 2007/1 MGF-360-19Ra/Rb will complex via hydrophobic interactions, and the N and C termini of 19 Ra and Rb are predicted to be within 18 Å of one another, as shown in Supplementary Figure S14B. 

#### 3.2.7. B263R__C315R

B263R has been predicted to be a TATA-box-binding protein [40], and C315R has been predicted to be at transcription factor II B (TFIIB) homolog [41]. The AFmult prediction that these two proteins interact provides another line of evidence that these two proteins are involved in viral transcription initiation. This predicted interaction appears to be driven predominately by favorable non-covalent electrostatic interactions, as shown in Supplementary Figure S15. 

#### 3.2.8. B175L__DP79L

B175L has been shown to block type-1 interferon-mediated response by interacting with cGAMP and STING to impair transduction of antiviral signals [42]. DP79L is an uncharacterized protein. 

#### 3.2.9. C475L__K421R

C475L predicted a poly(A) polymerase catalytic subunit [41], which is hypothesized to polyadenylate ASFV transcripts. K421R is an uncharacterized protein.

#### 3.2.10. CP80R__H359L

CP80R is the predicted RNA polymerase subunit 10, and H359L is the predicted RNA polymerase 3–11 (annotated RNA pol 3 in [41] fusion protein [41]. 

#### 3.2.11. D250R__D250R

D250R is an experimentally determined hydrolase and was his-tag purified as a monomer [43]. It is an mRNA decapping enzyme, and a D250R-MBP fusion was also purified as a monomer [44]. D250R can act to decrease both host and viral mRNA when over-expressed [45]. In addition to its monomeric state, it has been crystallized as a dimer, PDB structure 7DNT [46]. Furthermore, during infection, D250R has demonstrated constitutive expression and exhibits distinct subcellular localizations. Initially, it predominantly localizes to the endoplasmic reticulum (ER) in the early stages of infection. However, as the infection progresses, it tends to accumulate within the viral factories [45].

#### 3.2.12. B646L__B646L

B646L encodes for the major capsid protein of the ASFV. Structurally, p72 exists as a trimer [47] and has been visualized via cryo-EM. Although it scored 0.77 on the HAF screen, we ran it through AFmult and presented the results of this high-scoring dimer here. 

#### 3.2.13. F334L__F334L and F778R__F778R

The ASFV encodes a predicted ribonucleotide reductase composed of two small (F334L) and two large (F778R) subunits, as previously described in Section 3.2.3.

#### 3.2.14. B318L__B318L

B318L is an experimentally verified integral membrane trans-geranylgeranyl-diphosphate synthase [48,49]. While crystallization without the putative N-terminal transmembrane region was structured as a monomer, PDB 8HDL [50], the structure of the native protein has not been experimentally determined. 

#### 3.2.15. K196R__K196R

K196R is an experimentally verified thymidine kinase [51]. Deletions of this protein in ASFV Georgia 2007/1 cause viral attenuation but do not result in protection from the virulent parental strain [52]. The ASFV thymidine kinase (TK), a viral enzyme that synthesizes deoxynucleoside triphosphates [51], is not necessary for virus replication in cell cultures [53]. However, ASFV strains lacking the TK gene failed to replicate in swine macrophages [54]. Similarly, removing the TK gene from the ASFV Malawi isolate significantly attenuated the virus [54]. 

#### 3.2.16. QP383R__QP383R

QP383R is predicted to be an NifS-like PLP-dependent transferase [41]. It has been documented that it suppresses inflammatory reactions by blocking the activation of the AIM2 inflammasome [55]. Taking these findings together, QP383R is suggested to counteract the host’s innate immune response to ASFV infection by interacting with the central component, cGAS, in the cGAS-STING signaling pathways [55,56]. QP383R and QP509L have been deleted from the virulent strain ASFV CN/GS/2018 and caused a reduction in virulence but did not offer protection against lethal ASFV challenge [57].

#### 3.2.17. E165R__E165R

E164R is an experimentally verified deoxyuridine 5′-triphosphate nucleotidohydrolase (dUTPase) [49]. Crystal structures of it have been determined (PDB 6KY9 and 6KZ6), and it exists as a homo-trimer [58,59]. The deletion of QP383R in ASFV Georgia 2010 did not reduce virus replication or virulence in domestic pigs [60].

#### 3.2.18. A104R__A104R 

A104R__A104R is an experimentally verified DNA-binding protein that is closely associated with virus DNA in the virus particle and is important for viral DNA replication [61,62]. It has been crystallized and found to be a homodimer in complex with DNA (PDB 6LMJ, 6LMH) [63]. The deletion of A104R from ASFV Georgia2010 resulted in reduced virulence and lethality and elicited a strong virus-specific antibody response [64]. 

## 4. Discussion

In this work, we built on the approach used to identify yeast protein–protein interactions in Humphreys et al. 2021 [6] to identify PPIs in nucleocytoplasmic large DNA viruses, NCLDVs. We showed that the AlphaFold artificial intelligence structure inference algorithm outperformed RoseTTAFold when modeling viral protein interactions from the Vaccinia virus. This is likely due to the large pMSA sequence depth required by RoseTTAFold for accurate structure prediction, as this model relies more heavily on co-evolutionary signals for protein structure prediction than AlphaFold [6]. With the Humphreys et al. 2021 [6] lightweight AlphaFold model, the HAF, we were able to screen 23,871 and 19,110 PPIs in the Vaccinia virus and the ASFV, respectively, generating max contact probabilities for the vast majority of all pairwise combinations of viral proteins and only omitting a small number of large proteins that failed due to insufficient RAM. Running the high-scoring PPIs through a second pass of AlphaFold Multimer v2.3.2, AFmult, allowed us to increase our precision and generate more structurally accurate models. 

While our precision in the Vaccinia virus with our screen reached a maximum of around 20%, we believe this is a conservative estimate due to the lack of an exhaustive gold-standard PPI set for benchmarking. The only genome-wide PPI dataset in the Vaccinia virus comes from McCraith et al.’s 2000 work, in which a yeast-two hybrid was used. While this yields a wealth of valuable experimental evidence for the virus interactome, Y2H screens are by no means exhaustive due to the problems associated with the proteins being expressed in yeast. Some of these difficulties include the reliance on proteins to enter the nucleus, the fact that yeast does not glycosylate proteins, and other potential problems with both folding and post-translational modifications. Therefore, we believe the true precision of our computational screen to be higher than 20%. 

In the ASFV, no comprehensive experimental PPI screens have been conducted to date. This work identified seventeen potential ASFV PPI candidates. Five of these identified have been crystallized and corroborate our results. Seven interactions have direct experimental evidence for their existence or are backed up by strong predictive lines of evidence. Last, this screen identified the interactions I8L_I267L and B175L__DP79L, and there are no predictive lines of evidence for these interactions. I8L, I267L, and B175L all have been shown to be involved in modulating host immune response; see Section 3.2.1 and Section 3.2.7. 

We present these seventeen PPIs as candidates with strong lines of evidence for being biologically relevant. Experimental validation will be required to prove the existence and function of these protein pairs. We hope that the identification of these interactions will aid in the development of successful future candidates for affordable live attenuated vaccines against the African swine fever virus.

## Figures and Tables

**Table 1:** AlphaFold screen summary. Total PPIs include both homodimers and all pairwise combinations of heterodimers. The HAF is the Humphreys et al. 2021 [6] lightweight Alphafold v2.0. AFmult total are all models successfully generated with AlphaFold Multimer v2.3.2, and AFmult ≥ 0.8 are all AFmult models with ipTM + pTM scores of 0.8 or higher.

Virus	Total PPI	HAF ≥ 0.8	AFmult Total	AFmult ≥ 0.8
Vaccinia virus	23,871	592	572	34
ASFV	19,110	375	351	19

**Table 2:** ASFV-ASFV PPIs identified in our AlphaFold screen. See Supplementary Figures S11 and S12 for the structure and local confidence scores.

Protein A (Alternative Names)	Function A	Protein B (Alternative Names)	Function B	AFmult ipTM + pTM Score
CP530R (p62)	Encodes for polyprotein p62, which is cleaved into p15, p35, and p8 by S273R. p15 and p35 are found in the core–shell of the virion.	S273R (i6R) *	Cysteine protease that cleaves structural proteins pp220 and pp62. S273R has been shown to target various host proteins, leading to the inhibition of pyroptosis and cGAS-STING pathway-mediated type-I interferon (IFN) production.	0.80
I267L (k7L)	Experimentally verified early protein that inhibits RNA Pol-III-RIG-I-mediated antiviral response by interacting with Riplet and preventing the activation of RIG-I.	I8L (L8L)	Predicted to contain an Src homology 2 (SH2) domain. Early protein. Involved in virulence.	0.86
F334L (RR small)	Predicted ribonucleotide reductase (small subunit).	F778R (RR large)	Predicted ribonucleotide reductase (large subunit).	0.80
C475L	Predicted poly(A) polymerase catalytic subunit. Late protein.	K421R	Uncharacterized protein. Late protein.	0.89
CP80R	Predicted RNA polymerase subunit 10.	H359L (J1L)	Predicted RNA polymerase 3–11 fusion protein.	0.85
MGF_360-19Ra	Uncharacterized protein.	MGF_360-19Rb	Uncharacterized protein.	0.87
MGF_360-1La	Uncharacterized protein.	MGF_360-1Lb	Uncharacterized protein.	0.82
B175L	Antagonist of type-I IFN.	DP79L	Uncharacterized protein.	0.82
B263R	Predicted to be a TATA-box-binding protein. Early protein.	C315R	Predicted to be an initiation factor TFIIB homolog.	0.82
D250R *	Constitutively expressed hydrolase and mRNA decapping enzyme.	D250R *	Constitutively expressed hydrolase and mRNA decapping enzyme.	0.93
F334L (RR small)	Predicted ribonucleotide reductase (small subunit).	F334L (RR small)	Predicted ribonucleotide reductase (small subunit).	0.86
F778R (RR small)	Predicted ribonucleotide reductase (large subunit).	F778R (RR small)	Predicted ribonucleotide reductase (large subunit).	0.90
B646L (p72, p73) *	Major capsid protein.	B646L (p72, p73) *	Major capsid protein	0.91
B318L *	Integral membrane trans-geranylgeranyl-diphosphate synthase.	B318L *	Integral membrane trans-geranylgeranyl-diphosphate synthase.	0.87
K196R (TDK)	Thymidine kinase.	K196R (TDK)	Thymidine kinase.	0.85
QP383R (j11R)	Predicted to be an NifS-like PLP-dependent transferase. Represses inflammatory responses by inhibiting AIM2 inflammasome activation.	QP383R (j11R)	Predicted to be an NifS-like PLP-dependent transferase. Represses inflammatory responses by inhibiting AIM2 inflammasome activation.	0.84
E165R (K1R) *	Deoxyuridine 5′-triphosphate nucleotidohydrolase (dUTPase).	E165R (K1R) *	Deoxyuridine 5′-triphosphate nucleotidohydrolase (dUTPase).	0.83

* Proteins with experimentally determined crystal structures.

## Data Availability

Supplementary Data Files are available at Paper DOI. All scripts used in this work will be deposited to GitHub Global African swine research alliance (GARA), https://github.com/Global-ASFV-Research-Alliance/VirusPPIScreen (accessed on 4 June 2024). Any additional data from the manuscript will be provided upon request.

## Supplementary Materials

The following supporting information can be downloaded at https://www.mdpi.com/article/10.3390/v16071170/s1. S1—Supplementary Figures: Paired multiple sequence alignment, Pie chart of ASFV phyla represented in pre-filtered MSAs, Pie chart of Vaccinia Virus phyla represented in pre-filtered MSAs, ASFV-ASFV heterodimer paired Nseq, Vaccinia-Vaccinia heterodimer paired Nseq, ASFV-ASFV heterodimer paired Nseq80, Vaccinia-Vaccinia heterodimer paired Nseq80, Bioinformatic pipeline creating a MSA of homologues from an input viral protein, Paired-MSA, pMSA, generation and two-stage AlphaFold screening for PPI scoring, AlphaFold genome-wide PPI screening performance on Vaccinia virus, ASFV-ASFV heterodimer top hits, ASFV-ASFV homodimer top hits, CP530R__S273R AFmult predicted structure, Predicted MGF structures, and **.** B263R__C315R predicted structure. SD1—Supplementary Data 1, ASFV_PPI_data.csv; SD2—Supplementary Data 2, Vaccinia_PPI_data.csv; SD3—Supplementary Data 3, ASFV_AlphaFoldMultimer_models.tar.gz; SD4—Supplementary Data 4, Vaccinia_AlphaFoldMultimer_modles.tar.gz; SD5—Supplementary Data 5, Georgia-2007-LR743116_trans-rename.fa; SD6—Supplementary Data 6, uniprotkb_proteome_UP000000344_2024_01_18.fasta.
